# Bone resorption as a marker for delayed esophageal perforation post anterior cervical spine surgery: a retrospective analysis and call for increased vigilance

**DOI:** 10.3389/fmed.2024.1484712

**Published:** 2024-10-30

**Authors:** Hua Luo, Zhangfu Wang, Shuang Mi, Guangyong Yang, Wenjun Pan, Xingbing Feng, Zhenghua Hong

**Affiliations:** Department of Orthopedics, Taizhou Hospital of Zhejiang Province Affiliated to Wenzhou Medical University, Taizhou, China

**Keywords:** bone resorption, delayed esophageal perforation, anterior cervical spine surgery, esophageal repair, complication

## Abstract

**Purpose:**

Delayed esophageal perforation following anterior cervical spine surgery (ACSS) is a rare but serious complication. This study is to investigate the clinical characteristics, diagnostic approaches, and treatment outcomes of delayed esophageal perforation following ACSS, with a focus on the role of bone resorption around internal fixations as a potential diagnostic indicator.

**Methods:**

We retrospectively analyzed patients diagnosed with delayed esophageal perforation after ACSS from January 2010 to December 2023 and described their clinical characteristics, diagnostic approaches, and treatment outcomes. Through the analysis of the differences in the radiomics of patients, we identified the possible clinical signs of esophageal perforation and shared our experience in treating esophageal perforation.

**Results:**

A total of five patients met our criteria. All five patients exhibited bone resorption around their internal fixations on radiography. Although bone resorption typically suggests local infection, none of the patients showed clear signs of neck skin infection, leading us to suspect esophageal perforation as the underlying cause. Further diagnostic procedures including CT, MRI, esophagography, and endoscopy were crucial for confirming the diagnosis of delayed esophageal perforation and assessing its severity. All patients underwent surgical intervention involving implant removal and esophageal repair using a sternocleidomastoid muscle flap transfer. All patients recovered and were discharged after treatment, with no recurrence of symptoms during follow-up.

**Conclusion:**

Delayed esophageal perforation should be considered in patients with neck pain or nonspecific symptoms after ACSS, especially with bone resorption around internal fixations. Clinicians should maintain high vigilance and use multimodal imaging and endoscopy for timely diagnosis. Our study indicates a significant link between bone resorption and delayed esophageal perforation despite the limited number of cases. Highlighting this association aims to raise awareness and encourage further research. Larger studies are needed to validate our findings, improve clinical guidelines, and ultimately enhance patient outcomes in orthopedics.

## Introduction

Esophageal perforation is a rare complication after anterior cervical spine surgery (ACSS) that was first reported in 1985 (1) and has an incidence of 0.04 to 1.62%. Although the incidence of post ACSS esophageal perforation is relatively low, its potential consequences are extremely serious. Mild cases may involve incision infection and dysphagia, while severe cases may involve mediastinal infection, lung infection, sepsis, with a mortality rate of up to 20% (2, 3). Esophageal perforations are classified as early onset (within 30 days postoperatively) and late onset (more than 30 days postoperatively) (4, 5). Radiographs can indicate post-ACSS esophageal perforation through signs like subcutaneous emphysema, parapneumatosis, retropharyngeal-esophageal space widening, or fixations loosening (6–8). CT and MRI can reveal fluid accumulation, pneumatosis, or paravertebral region fixations loosening. Contrast-enhanced CT and MRI offer detailed esophageal morphology and may detect abscesses. However, diagnosing esophageal perforation typically relies on gastrointestinal endoscopy and esophagography, which have limitations like a 10% false-negative rate for esophagography and discomfort from contrast agents (9–11). Gastrointestinal endoscopy, though invasive, can miss small perforations near the incisors and risks delayed diagnosis leading to mediastinal infections (12, 13).

Given these issues, it would be ideal to identify a non-invasive examination to diagnose esophageal perforation in the early stage. Previous radiomics studies have focused on the accumulation of air in front of the cervical spine and the loosening of internal fixations, while there has been no research on bone resorption around the internal fixation. In the present study, we reviewed the patients with esophageal perforation diagnosed and treated in our hospital, explored the diagnostic value of radiomics for late-onset esophageal perforation, and shared our experience in the treatment of late-onset esophageal perforation.

## Materials and methods

This study was approved by the ethics committee of our hospital. We retrospectively analyzed patients with delayed esophageal perforation after ACSS treated in our hospital from January 2010 to December 2023. Data extracted from the patient records were age, sex, history of alcohol intake, history of smoking, medical comorbidities, date of surgery, indications for cervical surgery, cervical segments involved in surgery, history/symptoms of postoperative pharyngeal or esophageal injury, interval between surgery and onset of esophageal perforation, duration of follow-up, etc. Including their preoperative and postoperative imaging data.

### Surgical technique

After general anesthesia induction, longitudinal incisions were made, regardless of the original incision direction. The skin was incised to the deep fascia and separated medially to the prevertebral fascia, fully exposing internal fixation. All necrotic and infected tissue, along with internal fixation, were removed. Using preoperative esophagography and gastrointestinal endoscopy findings, the esophageal perforation was located and exposed, and the gastrointestinal tube was identified. Around the perforation, 1–2 mm of inflammatory tissue was trimmed, and the esophagus was closed with intermittent inversion sutures. If there was an esophageal diverticulum, it was excised, and esophagoplasty was performed to minimize esophageal tissue removal and stricture risk. The wound was irrigated, and the sternocleidomastoid muscle was freed, transposed, and fixed. Bone resorption cavity and perforation periphery were filled, and a drainage tube was inserted. Antibiotics continued until normalization of white blood cell count and C-reactive protein. Drainage tube removal occurred after 5 days, followed by liquid intake at 10 days and gradual diet resumption, avoiding hard foods for a month to prevent secondary esophageal injury.

## Results

Five patients (3 males and 2 females) with an average age of 44 years met our inclusion criteria, as detailed in Table 1. The average time from anterior cervical surgery to the onset of symptoms was 4.88 years. All five patients underwent surgery comprising implant removal and esophageal repair followed by sternocleidomastoid muscle flap transfer tamponade. Among the five patients, three had cervical spondylosis (1 patient had cervical spondylosis and 2 patients had cervical myelopathy), and the other two had cervical spine injuries due to trauma (1 with cervical hyperextension injury, and 1 with an unknown injury). Anterior cervical discectomy and fusion (ACDF) was performed in four patients, while one patient underwent C6 anterior cervical corpectomy and fusion (ACCF). Among them, one smoked, one had a history of hypertension, and a history of rectal cancer. One patient presented with dysphagia, three presented with neck and shoulder pain, and one was asymptomatic.

**Table 1:** Characteristics of included cases.

Case	Age (years) (at time of PEP surgery)	Sex	Medical history	Indication for cervical surgery	Cervical surgery	Level of PEP	Mechanism of PEP	Interval between PEP diagnosis (recurrence) and surgical treatment	Instrumentation removal	Posterior fixation (days before PEP repair surgery)	Type of flap	Outcome
Case 1	53	F	—	Cervical spondylosis	ACCF (C6)	MCS	Plate decubitus	10 Y	Plate removal	No	SCM	Resolution
Case 2	47	F	Hypertension, rectal cancer (moderately differentiated adenocarcinoma), uterine fibroids	Cervical spondylosis	ACDF (C4–6)	MCS	Plate decubitus	9 Y	Plate and partial (C5/6) cage removal	No	SCM	Resolution
Case 3	42	M	Smoking	Cervical spondylosis	ACDF (C5/6) + posterior single open-door laminoplasty (C4–7)	MCS	Plate decubitus	4 M	Plate and cage removal	Already present	SCM	Resolution
Case 4	53	M	—	Trauma	ACDF (C3/4 + C5/6) + posterior single open-door laminoplasty (C3–6)	MCS	Screw decubitus	5 Y	Prevail removal (C5/6)	Already present	SCM	Resolution
Case 5	28	M	—	Trauma	ACDF (C3–6)	CTJ	Screw malposition and migration	1 M	Plate and cage removal	No	SCM	Resolution

PEP, pharyngo-esophageal perforation; SCM, sternocleidomastoid muscle; MCS, midcervical spine; CTJ, cervicothoracic junction; ACCF, anterior cervical corpectomy and fusion; ACDF, anterior cervical discectomy and fusion.

Three of the five patients we treated had non-specific clinical manifestations comprising neck and shoulder pain. One patient was hospitalized for repeated treatments due to severe neck and shoulder pain and was incorrectly suspected of having mental and psychological problems, which caused great psychological damage to the patient. Among the five patients, two underwent esophageal diverticulectomy followed by esophagoplasty, and three underwent esophageal repair.

### Case 1

**Figure 1:**
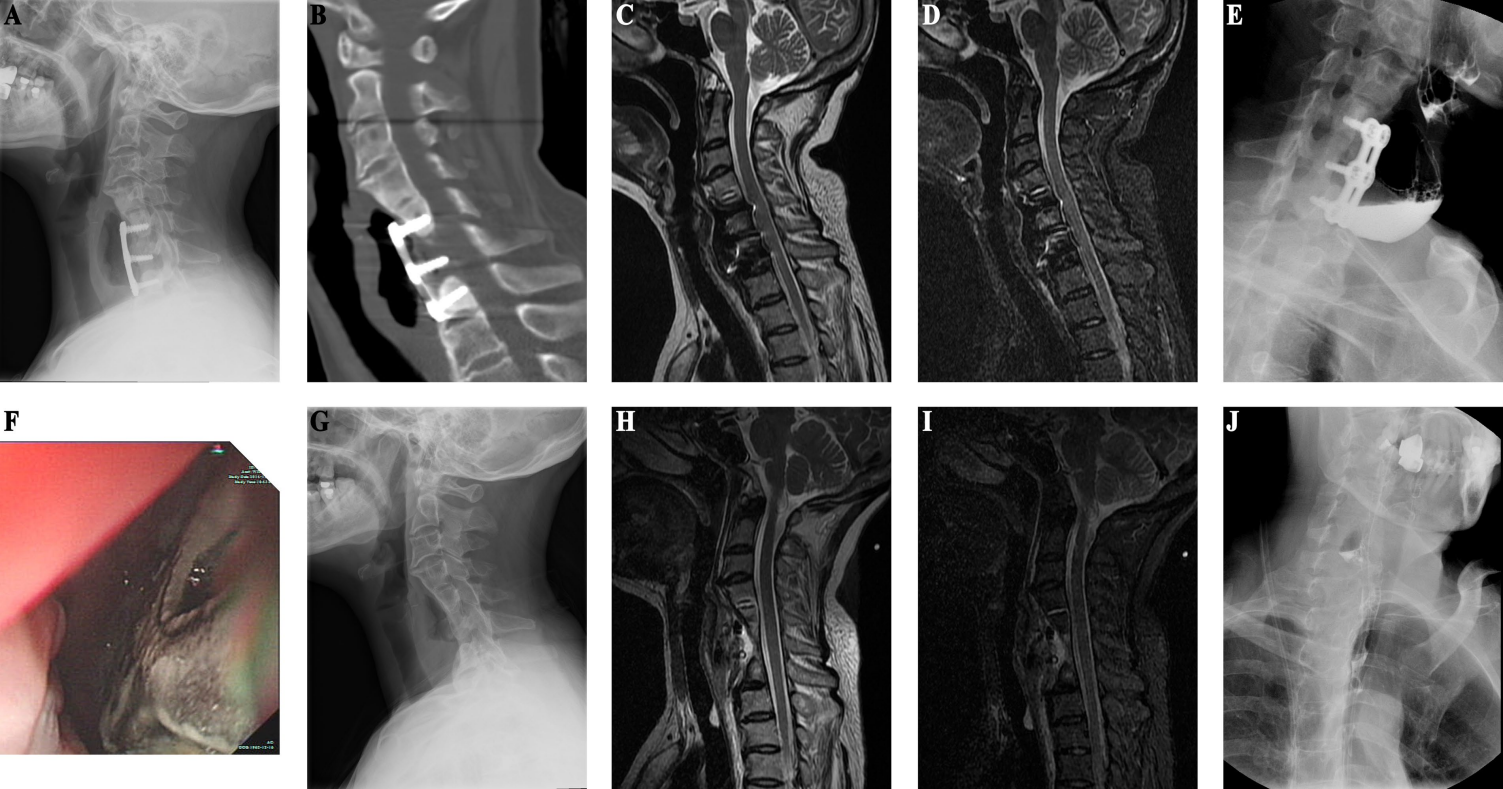
(Case 1) **(A)** Ten years post-cervical spine surgery, X-ray reveals bone resorption in the C5–7 vertebral bodies beneath the plate, with anterior plate pneumatisation. **(B)** CT shows partial osseous defect in the C5–7 vertebral bodies, accompanied by anterior soft tissue swelling and pneumatosis. **(C)** T2-weighted sequence shows anterior soft tissue pneumatosis and fluid accumulation. **(D)** STIR sequence depicts partial vertebral body loss and anterior high signal intensity. **(E)** Esophagography demonstrates extraluminal contrast extravasation into the cervical spine soft tissues, with a surrounding cavity measuring approximately 2.9 × 1.0 cm. **(F)** Upper endoscopy reveals esophageal diverticulum and a 5 cm posterior esophageal wall rupture defect, with visualization of the cervical spine plate. **(G)** Two years post-esophageal perforation repair, X-ray demonstrates preserved cervical spine physiological curvature, removal of cervical spine internal fixation, and localized C5–7 vertebral body bone loss. **(H)** Two-year postoperative cervical spine MRI T2-weighted images exhibit mixed anterior high and low signal intensity. **(I)** Postoperative STIR images illustrate partial vertebral body defects and anterior high signal intensity. **(J)** Postoperative esophagography reveals 2.0 × 0.6 cm intraluminal esophageal outpouching.

An adult patient with myelopathy underwent C6 ACCF in 2004. She developed right neck and shoulder pain and was suspected to have an esophageal perforation based on esophagography performed 6 months postoperatively. However, gastrointestinal endoscopy did not confirm the presence of an esophageal perforation. Over the next 7 years, she experienced persistent pain and sought medical attention multiple times without receiving a diagnosis. At 7 years postoperatively, she developed a mass in the right neck that was unable to be located during biopsy attempts. At 10 years postoperatively, she was diagnosed with lymphoma, which was later revealed to be caused by an esophageal perforation. On cervical radiography, the internal fixation of the cervical spine was structurally good, but there was a local bone defect in the C5–7 vertebrae under the steel plate (Figure 1A). CT showed a partial bone defect in the C5–7 vertebral bodies and swelling of the right paravertebral soft tissue with pneumata and effusion (Figure 1B). MRI showed localized swelling and effusion in the paravertebral soft tissues (Figures 1C,D). During esophagography, the contrast agent leaked into the soft tissues of the cervical spine to reveal a large surrounding cavity (Figure 1E). Gastrointestinal endoscopy revealed an esophageal diverticulum, a 5-cm-long ulcerative defect in the posterior wall of the esophagus, and a visible cervical steel plate (Figure 1F). Surgery revealed a large esophageal diverticulum with rupture, which was treated with esophagoplasty and muscle flap closure. Two years after the final esophageal perforation repair, X-ray shows preserved cervical spine curvature, C5-7 vertebral body bone loss, and removal of internal fixation (Figure 1G). MRI T2-weighted and STIR images demonstrate mixed anterior high and low signal intensity with partial vertebral body defects (Figures 1H,I). Esophagography shows a 2.0 x 0.6 cm intraluminal esophageal outpouching (Figure 1J). After 8 years of follow-up, her symptoms had resolved and the wound had healed.

### Case 2

**Figure 2:**
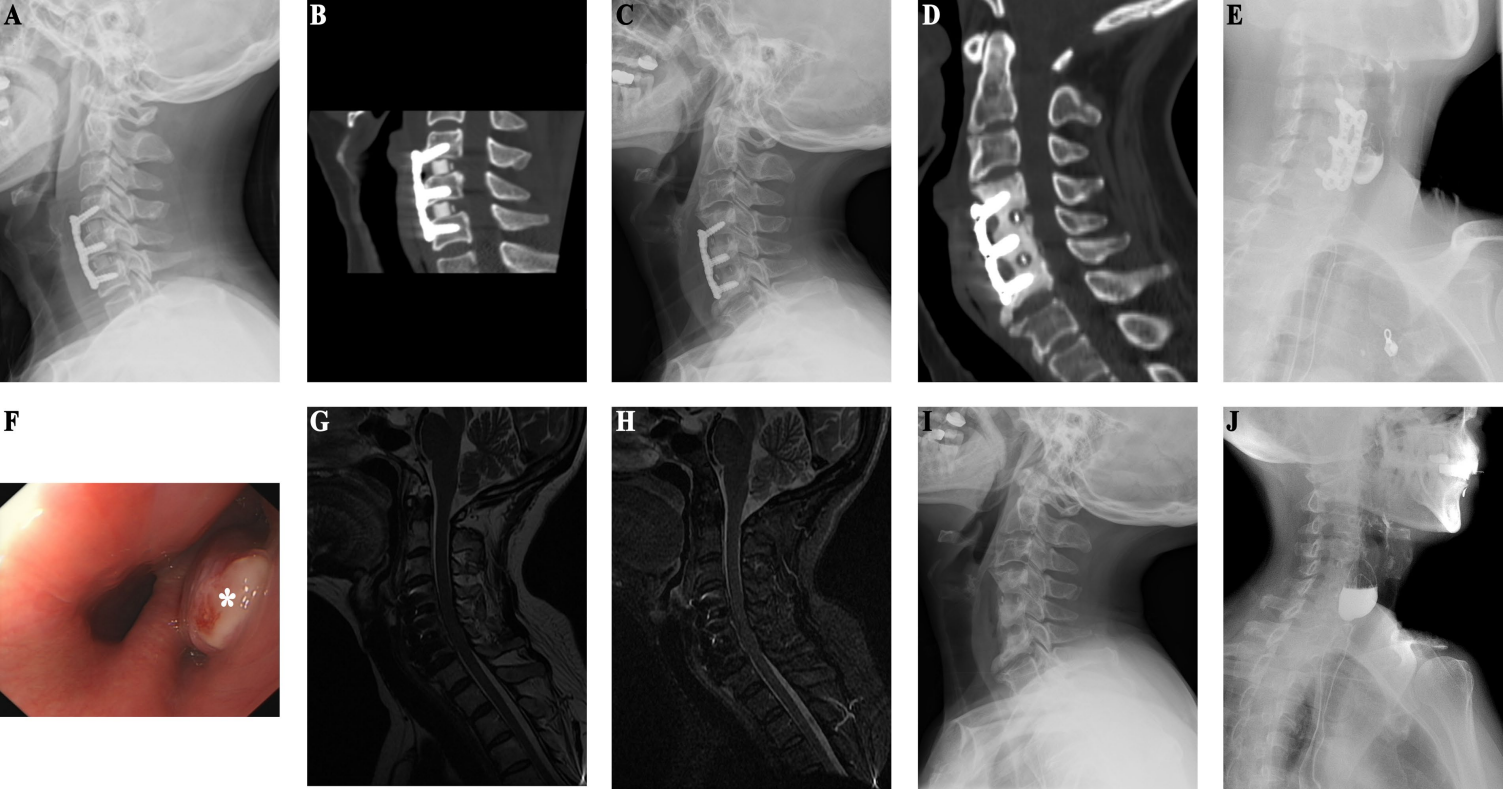
(Case 2) **(A)** One-year postoperative follow-up X-ray after ACDF shows preserved physiological curvature of the cervical spine with no signs of loosening of internal fixation. **(B)** One-year post-ACDF CT reveals no evidence of bone resorption beneath the plate. **(C)** X-ray findings nine years post-ACDF demonstrate significant bone absorption and increased density in the vertebral bodies at the C4–6 level. **(D)** CT findings are consistent with X-ray results. **(E)** Esophagography indicates residual contrast material in a localized pouch-like formation in the upper segment of the esophagus, measuring approximately 3.3 × 2.7 cm. **(F)** Endoscopic examination reveals a bulge in the esophagus approximately 15 cm distal to the incisors (as indicated by * in the diagram). **(G)** T2-weighted sequence shows high mixed signal intensity anterior to the vertebral bodies at the C4–6 level. **(H)** STIR sequence demonstrates pronounced anterior soft tissue swelling. **(I)** Half-month post-esophageal perforation repair X-ray shows removal of internal fixation devices at the C4–6 level, with localized vertebral body defect. **(J)** Postoperative esophagography reveals residual contrast material in a localized pouch-like formation in the upper segment of the esophagus, measuring approximately 2.8 × 1.9 cm.

Patient in their 40s who underwent C4–6 ACDF surgery 9 years ago for cervical spondylosis was admitted for intestinal tumor evaluation (Figures 2A,B). Due to her surgical history, she underwent cervical spine imaging that revealed thickened prevertebral soft tissue, bone resorption under the steel plate, and marginal osteosclerosis (Figure 2C). CT confirmed the presence of bone resorption and soft tissue thickening. There was also increased bone density at C4–6 (Figure 2D). An esophageal perforation was suspected. MRI showed soft tissue swelling in front of the vertebral C4–6 (Figures 2G,H). Gastrointestinal endoscopy revealed esophageal bulging (Figure 2F), and esophagography confirmed diverticulum formation (Figure 2E). The patient underwent surgical removal of the diverticulum and internal fixation, with esophagoplasty and muscle flap closure. Half a month after esophageal perforation repair, X-ray shows removal of C4-6 internal fixation with a localized vertebral defect (Figure 2I), and esophagography reveals a 2.8 x 1.9 cm residual contrast pouch in the upper esophagus (Figure 2J). She recovered well and had no discomfort during 4 years of follow-up, suggesting that she had experienced an occult esophageal perforation.

### Case 3

An adult patient underwent C5/6 ACDF to treat unsteady walking and limb numbness. Postoperative MRI showed spinal cord compression and soft tissue swelling, and the patient underwent posterior single open-door laminoplasty (C4–7) at a local hospital. He subsequently developed right neck redness, swelling, and pain, which was diagnosed as a cervical anteribrassal abscess requiring debridement. However, the wound did not heal and food debris leaked. Upon admission to our hospital, imaging revealed soft tissue swelling, bone absorption under the steel plate, and a bone defect below the fixation. Esophagography and endoscopy confirmed esophageal perforation. Surgery comprised internal fixation removal, neck wound expansion, esophageal repair, and muscle flap closure. The patient fully recovered, with no recurrence of symptoms during 2 years of follow-up.

### Case 4

**Figure 3:**
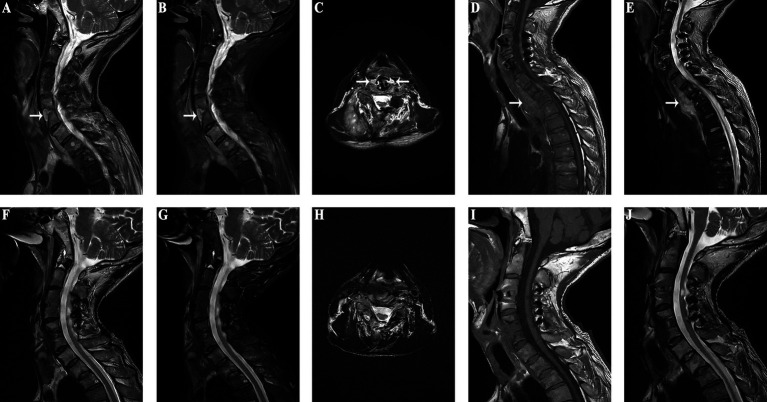
(Case 4) **(A–C)** An inflammatory mass anterior to the C5/6 vertebrae is observed preoperatively (as indicated by the white arrow in the diagram). **(D)** MRI of the thoracic spine reveals low signal intensity anterior to the C7-T2 vertebrae on T1-weighted images (as pointed by the arrow in the diagram). **(E)** MRI of the thoracic spine shows high signal intensity anterior to the C7-T2 vertebrae on T2-weighted images (as indicated by the arrow in the diagram). **(F–H)**. Postoperative MRI shows no inflammatory mass in front of the cervical spine. **(I)** Resolution of the low signal intensity anterior to the C7-T2 vertebrae on T1-weighted images. **(J)** Disappearance of the high signal intensity anterior to the C7-T2 vertebrae on T2-weighted images.

An adult patient was paralyzed due to quadriplegia from trauma and underwent C3/4 and C5/6 ACDF, TN, along with posterior cervical single-door surgery. Although the limb function was significantly improved postoperatively, at 5 years postoperatively the patient developed worsening neck and shoulder pain with recurrent fever, suggestive of a cervical esophageal perforation. Four months of antibiotic treatment resulted in no improvement. Upon admission to our hospital, imaging revealed bone resorption around the C5 and C6. Given the clinical suspicion of an esophageal perforation, the patient was admitted for anterior cervical surgery. Because of the intermittent fever, low protein, and elevated inflammatory markers, nutritional support was initiated. Subsequent investigations, including MRI, esophagography, gastrointestinal endoscopy confirmed an esophageal perforation. MRI suggests mass in front of C5/6 vertebral body (Figures 3A–C), and abnormal signal in front of C7 to T2 vertebral bodies suggests potential mediastinal infection (Figures 3D,E). After stabilizing the patient’s condition, surgery revealed significant vertebral body absorption and esophageal rupture. Internal fixation removal (C5/6), esophageal repair, and sternocleidomastoid muscle flap occlusion were performed. Postoperative follow-up MRI shows that the inflammatory mass in front of the cervical spine has resolved (Figures 3F–H). Follow-up MRI performed 1 month postoperatively showed resolution of the mediastinal abnormalities (Figures 3I,J), and the patient remained asymptomatic with no abnormal imaging findings at 1 year postoperatively.

### Case 5

Patient in their 20s who was paralyzed due to trauma underwent C3–6 ACDF. Postoperatively, he developed a red, swollen incision that was treated as a neck abscess and underwent repeated debridement. One month later, he required removal of loose screws. Despite initial improvement, the neck symptoms recurred 1 month after screw removal and resolved with anti-infection therapy. One year later, the neck redness and swelling recurred and radiography revealed a displaced fusion device and bone resorption. Gastrointestinal endoscopy revealed island-like mucosal redness, while an esophagography was negative. During surgery, pus drainage revealed an abscess communicating with the C5–6 space, with pus around the steel plate and bone surface. The esophageal perforation was treated with fixation and esophageal repair. There has been no recurrence in 9 years.

## Discussion

Only one of our five patients developed a secondary esophageal perforation caused by a screw loosening and puncturing the esophagus, while the remaining four patients did not have any loosening of the internal fixations. Furthermore, only one of the five patients presented with subcutaneous pneumatosis. However, the radiographs showed bone resorption around the internal fixations in all five patients. Our study is the first to suggest an association between bone resorption around the internal fixation and the occurrence of esophageal perforation following anterior cervical spine surgery. Additionally, we experienced a case in which the patient had no clinical signs but had an occult esophageal perforation. If bone resorption around the fixation is shown on radiography during follow-up after anterior cervical surgery, CT should be performed to determine the degree and extent of bone resorption, and MRI should be performed to assess the soft tissue swelling around the fixation.

The interface between the bone and the fixed plate is often the site of infectious complications (14). Because the attachment of the steel plate may reduce blood circulation, bacteria can grow and form biofilms that are difficult to remove (15, 16). We only suspected that the patient in case 1 may have an esophageal perforation after it had ruptured. Our experience in diagnosing and treating case 1 indicated that bone resorption around the internal fixation after anterior cervical spine surgery may serve as a radiological indicator of an esophageal perforation. All of the four subsequent patients with bone resorption around the internal fixation after surgery via the anterior cervical approach underwent gastrointestinal endoscopy and esophagogram and were diagnosed with secondary esophageal perforation. During gastrointestinal endoscopy, we strongly recommend that the examining doctor pays attention to the part of the esophagus that is about 15 cm away from the incisors (i.e., the esophagus near the anterior cervical steel plate) to reduce the chance of missing the diagnosis of esophageal perforation. A previous retrospective study showed that only 32 of 44 patients with esophageal perforation had esophageal injury suggested by imaging (17). Therefore, the diagnosis of esophageal perforation requires comprehensive consideration of multiple aspects, including imaging and clinical signs and symptoms. Patients with suspected esophageal perforation require repeated tests to avoid diagnostic delays. The patient in case 2 with an occult esophageal perforation had no discomfort except for dysphagia about 2 years postoperatively, and the bone resorption under the plate was found during a routine radiographic examination of the cervical spine.

All five of the patients in the present case series developed esophageal perforation at the C5/6 level. The first esophageal stenosis is located at the proximal end, at the level of the lower border of C6, about 15 cm from the central incisors. At this level, the bulging of the esophagus at the junction with the distal narrow portion is more likely to cause the edge of the steel plate to rub around the esophagus. In addition, while the digestive tract has serous and submucosal layers that contain tension-resistant collagen and elastic fibers, the esophagus does not have a serous layer, making it more susceptible to injury (18). The anatomical position of Killian’s triangle, located between C4 and C6, represents a weak area of the esophagus, which further contributes to the risk of esophageal perforation (19). Furthermore, the esophagus is very easily injured and perforated in patients with a cervical bone spur and neck hyperextension.

The successful management of esophageal perforation depends on prompt recognition of symptoms and immediate treatment. It has been reported that an early esophageal perforation smaller than 1 cm can be treated solely by fasting and regularly changing the wound dressing, while those larger than 1 cm require surgical treatment (20). Late esophageal perforations require direct surgical repair, and muscle valve metastasis and occlusion are performed to reduce dead space, increase the blood supply around the esophagus, promote esophageal healing, and prevent esophageal re-injury (21). The reconstruction phase involves direct multilayered interrupted inverting suture of at least the mucosal and muscular layers to reduce the risk of pharyngeal esophageal stricture following pharyngeal esophageal perforation (3, 5, 22). For patients with large perforations or intraoperative problems with tissue quality, the sternocleidomastoid muscle or pectoralis major muscle flap should be inserted between the sutured pharyngeal esophageal wall and the vertebral plane. Of our five patients, two underwent esophagoplasty and three underwent esophageal repair; however, all five patients underwent sternocleidomastoid muscle flap tamponade. In addition, any esophageal diverticulum should be removed and the esophagus repaired to prevent surgical failure. Muscle flaps protect the esophagus from compression or injury, provide blood supply, increase the concentration of antibiotics, and fill the dead space to increase the likelihood of surgical success. The buccopharyngeal fascia descends along the posterior aspect of the esophagus and extends outward to the carotid sheaths, delineating a boundary between the esophagus and the prevertebral fascia. These layers of fascia establish a paraesophageal space and a retroesophageal space, forming compartments that can facilitate the spread of cervical infections into the mediastinum (23).

### Strengths and limitations

Previous studies have suggested that esophageal perforation may be diagnosed based on the presence of imaging features such as parapneumovertebral pneumospondyla, widening of the retropharyngeal-esophageal space, or displacement of internal fixation, with no mention of lamellar bone resorption. This study is the first to propose that bone resorption beneath the plate can be used as a potential marker for esophageal perforation following ACSS. Radiography is the simplest and most non-invasive method with which to detect bone resorption. If radiography shows bone resorption under the steel plate, the diagnosis of esophageal perforation can be confirmed through a series of radiomics such as CT, MRI, gastrointestinal endoscopy, and esophagography. We acknowledge that our case series, limited to five patients, may not provide robust evidence. However, the implications of our findings are significant. By highlighting the potential link between bone resorption around internal fixations and delayed esophageal perforation, we aim to raise awareness and encourage further research in this area. Future studies with larger cohorts are necessary to validate our observations and provide higher levels of evidence. This will ultimately contribute to better clinical guidelines and improved patient outcomes in the field of orthopedics.

This study also has some limitations. First, this was a retrospective study of only five patients. A large, multicenter study is warranted to confirm the value of bone resorption under the plate in the diagnosis of esophageal perforation and to identify the incidence of and the risk factors for esophageal perforation. However, as esophageal perforation is a rare complication and there is no database of similar cases, it may be difficult to accumulate a large number of patients. Second, although lamellar bone resorption may indicate the possibility of an esophageal perforation, such bone resorption is often representative of advanced esophageal perforation. Therefore, it is necessary to identify a method with which to diagnose esophageal perforation in the early stage.

## Conclusion

Delayed esophageal perforation should be considered in patients with neck pain or nonspecific symptoms after ACSS, especially when bone resorption around internal fixations is present. Our findings suggest that bone resorption, typically an indicator of local infection, may also indicate esophageal perforation even without obvious infection symptoms. Clinicians should therefore maintain a high level of suspicion and use multimodal imaging and endoscopic evaluation for timely and accurate diagnosis. Although our study sample is small, the potential link between bone resorption and delayed esophageal perforation is significant. Highlighting this association aims to raise awareness and encourage further research. Future studies with larger cohorts are needed to validate our observations and provide stronger evidence, ultimately leading to better clinical guidelines and improved patient outcomes in orthopedics.

## Data Availability

The raw data supporting the conclusions of this article will be made available by the authors, without undue reservation.
